# ENABLE 2017, the First European PhD and Post-Doc Symposium. Session 2: The OMICS Revolution

**DOI:** 10.3390/biom8040116

**Published:** 2018-10-17

**Authors:** Gianmarco Di Mauro, Ambra Dondi, Giovanni Giangreco, Alexander Hogrebe, Elja Louer, Elisa Magistrati, Meeli Mullari, Gemma Turon, Wouter Verdurmen, Helena Xicoy Cortada, Sanja Zivanovic

**Affiliations:** 1 Institute for Research in Biomedicine (IRB Barcelona), The Barcelona Institute of Science and Technology, Baldiri Reixac 10, 08028 Barcelona, Spain; gemma.turon@irbbarcelona.org (G.T.); sanja.zivanovic@irbbarcelona.org (S.Z.); 2 European School of Molecular Medicine (SEMM), via Adamello 16, 20139 Milano, Italy; ambra.dondi@ieo.it (A.D.); giovanni.giangreco@ieo.it (G.G.); elisa.magistrati@ifom.eu (E.M.); 3 Novo Nordisk Foundation Center for Protein Research, University of Copenhagen, Blegdamsvej 3B, DK-2200 Copenhagen N, Denmark; alexander.hogrebe@cpr.ku.dk (A.H.); meeli.mullari@cpr.ku.dk (M.M.); 4 Radboud Institute for Molecular Life Sciences (RIMLS), Radboud University Medical Center, Geert Grooteplein 28, 6525 GA Nijmegen, The Netherlands; elja.louer@radboudumc.nl (E.L.); wouter.verdurmen@radboudumc.nl (W.V.); helena.xicoy@radboudumc.nl (H.X.C.)

**Keywords:** biomedicine, symposium, OMICS, proteomics, SWATH-MS, single-cell sequencing, metabolomics, genomics, lipidomics

## Abstract

The European Academy for Biomedical Science (ENABLE) is an initiative funded by the European Union Horizon 2020 program involving four renowned European Research Institutes (Institute for Research in Biomedicine—IRB Barcelona, Spain; Radboud Institute for Molecular Life Sciences—RIMLS, The Netherlands; Novo Nordisk Foundation Center for Protein Research—NNF CPR, Denmark; European School of Molecular Medicine—SEMM, Italy) and an innovative science communication agency (Scienseed). With the aim of promoting biomedical science of excellence in Europe, ENABLE organizes an annual three-day international event. This gathering includes a top-level scientific symposium bringing together leading scientists, PhD students, and post-doctoral fellows; career development activities supporting the progression of young researchers and fostering discussion about opportunities beyond the bench; and outreach activities stimulating the interaction between science and society. The first European PhD and Post-Doc Symposium, entitled “Breaking Down Complexity: Innovative Models and Techniques in Biomedicine”, was hosted by the vibrant city of Barcelona. The scientific program of the conference was focused on the most recent advances and applications of modern techniques and models in biomedical research and covered a wide range of topics, from synthetic biology to translational medicine. Overall, the event was a great success, with more than 200 attendees from all over Europe actively participating in the symposium by presenting their research and exchanging ideas with their peers and world-renowned scientists.

## 1. Introduction

**Figure 1:**
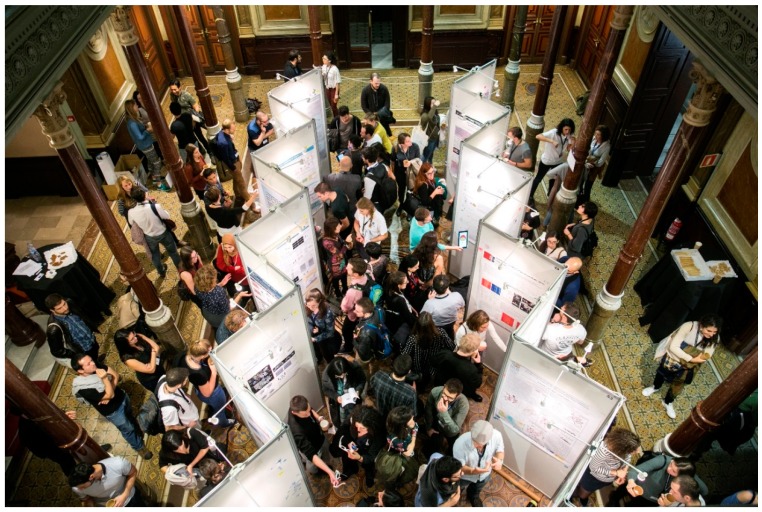
Attendees at ENABLE 2017.

Funded by Horizon 2020, the European Academy for Biomedical Science (ENABLE) project celebrates European research and brings together PhDs and postdocs from all over Europe via activities organized by volunteers and coordinators from the four host institutes—Institute for Research in Biomedicine (IRB) in Barcelona, European School of Molecular Medicine (SEMM) in Milan, Radboud Institute for Molecular Life Sciences (RIMLS) in Nijmegen, and Center for Protein Research (CPR) in Copenhagen. In November 2017, the first ENABLE conference took place in Barcelona, Spain. The organization of this conference started almost two years earlier and involved a group of 35 volunteer PhD students and postdocs from the four aforementioned research institutes and the support of institute coordinators and the innovative science communication agency, Scienseed. Like many young scientists nowadays, we felt isolated in our own research areas and wanted to build networks beyond our own fields. This is why we launched ENABLE and what the first ENABLE conference also achieved: it involved young scientists opening the academic world from within and promoted crosstalk between disciplines, collaboration with industry, and communication with society at large. The conference in Barcelona was a huge success, with the participation of 272 young researchers from more than 25 countries within the European Union (EU) and beyond (Figure 1). Companies were also thrilled by our approach, as reflected by our 10 sponsors, who provided more than 60 travel grants, and the more than 20 organizations that were present at our Career Day.

The scientific part of the ENABLE conference was a symposium entitled “Breaking Down Complexity: Innovative Models and Techniques in Biomedicine”. It comprised four sessions, spanning from molecular research to clinical research and potential new therapies. In each session, two distinguished keynote speakers presented their research and gave an overview of their work and the directions their fields are taking. Each keynote lecture was followed by two outstanding presentations by postdocs or PhDs chosen from among the 272 participants. In addition, 100 posters from a range of biomedical fields were presented by the participants, thus facilitating additional discussion between young researchers with diverse backgrounds. Last but not least, the conference also drew society into the discussion by organizing public debates with experts in the field, inviting school children to take part in scientific problem-solving activities at IRB Barcelona, and giving 24 participating young scientists the opportunity to present their work to the public.

## 2. Session 2: The OMICS Revolution

**Prof. Johan Auwerx** (Principal investigator, École Polytechnique Fédérale, Lausanne, Switzerland) was the first keynote speaker of the “The OMICS Revolution” session on the first day of the ENABLE scientific symposium. He specializes in studying metabolism in health, aging, and disease using approaches from molecular physiology combined with systems genetics and proteomics. His work in the past uncovered how diet, exercise, and hormones modulate metabolism by altering gene expression. His latest research focuses on characterizing an inbred population of BXD mice, resulting in more than 300 available genetic strains. The fact that these animals are inbred but still yield more than five million genetic sequence variations allows the mapping of phenotypic traits back onto their genetic origin. The integration of genetic data with those acquired via transcriptomics, proteomics, metabolomics, lipidomics, metagenomics, and phenomics revealed unexpected phenotypic correlations within the mouse population and underlined the immense strength of such multi-omics research strategies. In particular, the study revealed that female mice, which are often not used in biological research, can show significantly different molecular phenotypes in response to distinct stimuli or environments compared to male mice. Furthermore, the BXD panel showed a great variety in longevity, which was surprisingly prolonged by supplying a high-fat diet at high age. Finally, deep coverage proteomics analysis using sequential window acquisition of all theoretical fragment ion spectra mass spectrometry (SWATH-MS) in collaboration with Ruedi Aebersold, the second keynote speaker of the OMICS session, showed that the proteome changed dynamically depending on the kind of diet the mice received. This approach allowed the group to identify super complexes involved in the regulation of metabolism. All in all, Prof. Auwerx demonstrated the importance of using model organisms to answer biomedical questions, as they allow experiments to be performed on animals with a defined genetic heritage and in a controlled environment.

**Guido van Mierlo** (PhD student, Radboud Institute for Molecular Life Sciences, Nijmegen, The Netherlands) presented the first short talk of the OMICS session, in which he addressed the use of proteomics to study the ground state pluripotent epigenome of embryonic stem cells (ESCs). Recent advances in stem cell research have allowed the induction of this ESC ground state by using only two kinase inhibitors (2i). By applying integrative mass spectrometry to interrogate chromatin-associated proteins and histone modifications of 2i cells, Guido discovered that the protein complex PRC2 exerts a protective role over the ground state pluripotent epigenome.

**Dr. Mireya Plass** (Post-Doctoral researcher, Berlin Institute for Medical Systems Biology, Max-Delbrück Center for Molecular Medicine, Berlin, Germany) closed the first part of the OMICS session by presenting her results on generating a cell-type atlas with lineage tree reconstruction of whole adult animals by means of single cell sequencing. This was possible by combining drop-seq analysis and the development of new computational tools for lineage reconstruction with RNAi knock-down experiments to kill proliferating cells in the planarian *Schmidtea mediterranea*. These animals have a large pool of adult pluripotent stem cells that continuously regenerate the whole body and therefore contain cells in all possible differentiation stages. Her research revealed progenitors for most cellular lineages and identified new gene sets likely containing genes involved in the differentiation of stem cells to all the identified cell types.

**Prof. Ruedi Aebersold** (Principal investigator, University of Zurich, Switzerland and ETH Zurich, Zurich, Switzerland), one of the most renowned scientists in the mass spectrometry (MS) field, gave the second keynote talk of the OMICS session. He offered food for thought by showing how combining different omics data and adding prior knowledge about interactions in the cell can further our understanding about biology and disease. The key methodology to pursue this goal, SWATH-MS, has been developed in the Aebersold lab and is commonly used by this group, as well as many others. SWATH-MS combines data-independent acquisition (DIA), an unbiased way of acquiring MS data, and a novel way of analyzing the acquired raw data. Very briefly, DIA, in contrast to traditional data dependent acquisition (DDA), does not make analytical “on-the-go” choices based on the data being acquired during mass spectrometry scans. Instead, DIA attempts to acquire data for all possible analytes present in the sample at the same time. This method was also used for a study described in this talk, where 14 cell variants of HeLa from different labs were analyzed. The study showed that the slight differences that have arisen in these HeLa variants over time translate into much greater variation at the proteome level, where the variants were shown to be very different from each other. This is an example of how omics data gathered at different levels in the cell can complement each other and add different levels of information. This was additionally shown with a study where genomic data, proteomic data, and prior knowledge of protein-protein interactions were combined. In short, this study showed that changes in individual genes can lead to significant changes at the proteome level and in the disease phenotype, by disrupting the formation of complexes that involve the protein encoded by the mutated gene. In this regard, an individual mutation in a gene can disrupt the functions of many other proteins and have severe effects on the cell, thereby also illustrating how small genotypic changes can lead to a larger variation at the proteome level. The take-home message from the talk was that MS is a powerful tool with which to study proteins in an unbiased way, and the giant datasets themselves give rise to new bioinformatic challenges regarding how the data can be analyzed and integrated with additional contextual information.

**Nicolò Caporale** (PhD student, European Institute of Oncology, Milan, Italy) talked about a study focusing on the effect of endocrine-disrupting chemicals (EDCs) on development. EDCs have previously been linked to neurodevelopmental disorders, and the study followed the effect on compounds on a cohort and in various model systems ranging from organoids to stem cells. In a mother–child pregnancy cohort, 2 different mixtures of EDCs tested showed adverse outcomes. The in vitro studies in their model systems identified many genes whose regulation was altered after exposure to EDCs. With these discoveries, Nicolò and his colleagues have come a step closer to understanding the molecular pathways underlying these adverse effects.

**Flavia Greiffo** (PhD student, Helmholtz Zentrum, Munich, Germany) gave a talk about idiopathic pulmonary fibrosis (IPF), a fibroproliferative lung disease with irreversible loss of lung function. The researchers’ study found that myeloid-derived suppressor cells (MDSCs) are increased in number and functionally active, and that they reflect disease status in IPF, in cross-sectional, and longitudinal analysis. MDSCs are pathologically activated immature myeloid cells that suppress immune responses in cancer, autoimmunity, and other inflammatory conditions. Monocytic MDSCs are the predominant subtype in IPF, and yet differences between mature monocytes and monocytic MDSCs and their interaction in IPF have not been explored. In their study, label-free quantitative MS-analysis was used on monocytes and MDSCs isolated from the blood of ten IPF patients. In total, the researchers identified and quantified more than 7000 proteins from these cell types and found interesting differences between the two cell types. In addition, they also studied the receptors and ligands expressed by both cell types. These findings might lead to the identification of future therapeutic targets in IPF.

## 3. Conclusions and Future Perspectives of ENABLE

The first ENABLE conference was a success: 35 PhD and postdoc volunteers from four European research institutes, with support from the institute coordinators and Scienseed, organized an event in which 272 young researchers from over 25 countries presented and shared their science and experiences in scientific talks, poster sessions, master classes, general public talks, and evening activities. More than 60 attendees were given the opportunity to participate through the award of a travel grant funded by one of our 10 sponsors. The symposium, entitled “Breaking Down Complexity: Innovative Models and Techniques in Biomedicine”, was created to cover a broad range of topics in biomedical research, to encourage participation and the exchange of ideas, and to promote future collaborations among young scientists.

In order to include multiple research areas, there was a scientific program with four sessions. The first, entitled “Building the Foundations of Biology: Synthetic and Cellular Research”, featured Prof. Martin Hanczyc (University of Trento, Trento, Italy) and Prof. Elaine Fuchs (The Rockefeller University, New York, NY, USA) as keynote speakers and included short talks on DAPK regulation, nanoscale redistribution of NMDAR in autoimmune encephalitis, targets associated with metabolic reprogramming in hematological malignancies, and the mechanisms regulating aortic arch development. The second session, “The OMICS Revolution: Understanding the Layers of Life”, featured Prof. Johan Auwerx (École Polytechnique Fédérale, Lausanne, Switzerland) and Prof. Ruedi Aebersold (Eidgenössische Technische Hochschule (ETH), Zurich, Switzerland) as keynote speakers and included short talks on proteomics to study the role of polycomb repressive complex 2 (PRC2) in embryonic stem cells, single-cell sequencing to reconstruct the cell lineages of whole adult animals, the effects of endocrine-disrupting chemicals on development, and on quantitative proteomics to study fibrotic networks. The third session, “In Vitro to in Vivo: Modeling Life in 3D”, featured Prof. Kristina Havas Cavalletti (FIRC Institute of Molecular Oncology (IFOM), Milan, Italy) and Dr. Kim Jensen (Biotech Research and Innovation Centre (BRIC), Copenhagen, Denmark) as keynote speakers and included short talks on gut vascular barrier disruption and type 2 diabetes, the link between metabolic dysfunction and immune complications in lysinuric protein intolerance, the role of obesity in the development of acute promyelocytic leukemia, and the TGF-β pathway in colorectal cancer metastasis. The fourth and final session, “From Discovery to Cure: The Future of Therapeutics”, featured Prof. Eytan Ruppin (University of Maryland, Center for Bioinformatics and Computational Biology (CBCB), College Park, MD, USA) and Prof. Christian Brander (IrsiCaixa, Barcelona, Spain) as keynote speakers and included short talks on the role of miR27a as a tumor suppressor, photodynamic cancer therapy, real-time in vivo monitoring of transplanted islets, and a high-density lipoprotein nanodisc for the potential treatment of cerebral β-amyloidosis.

The success of the event was confirmed by the satisfaction scores (out of 5) given by the participants. In this regard, they gave the 2017 ENABLE symposium 4.4, the general topic of the symposium 4.1, and the keynote talks 4.4. The favorite part of the symposium was “Tapas with the Speakers” (score of 4.5), an activity that allowed the participants to interact with the keynote speakers in an informal setting while enjoying some typical Spanish food. The satisfaction of the attendees was also reflected by comments made on the evaluation form, such as “The option of travel grants is amazing and the general idea of the symposium is great. Great speakers, amazing food, nice event. Congratulations”, “I think that the ENABLE project is an amazing idea. It is a little bit different than other conferences because of the career day. The fact that it was organized by PhD students is really interesting!”, and “On the all, the symposia was super nice and it was extremely great to really discuss science on a reality level. No one wanted to show off or pretended to be the best scientist in the world and this was awesome”.

To conclude, the symposium brought together renowned scientists with young scientists and can be considered a huge success. The enthusiasm of the participants and the positive feedback received after the event underscore this notion and indicate that the ENABLE conference series has got off to an excellent start, with all eyes now focused on the 2018 event in Copenhagen.

The second symposium of the ENABLE series will be hosted by the Novo Nordisk Foundation Center for Protein Research (CPR, University of Copenhagen, Denmark), one of the partner institutions of the ENABLE consortium. It will take place 6–9 November 2018 at the Maersk Tower in Copenhagen.

Entitled “The Promise of Future Medicine: From Research to Therapy”, the symposium will explore state-of-the-art biomedical research from basic science to clinical practice and patient outcome. By bringing together 300 PhD students and postdocs, as well as nine eminent keynote speakers from diverse research fields, the next ENABLE symposium seeks to foster a multidisciplinary environment and crosstalk between biomedical disciplines. The following speakers have already confirmed their participation: Helen Lee (Cambridge University, Cambridge, UK); Giuseppe Testa (European Institute of Oncology, Milan, Italy); Nazneen Rahman (Institute of Cancer Research, London, UK); Klaus Pantel (Institute of Tumour Biology, University Medical Centre Hamburg-Eppendorf, Hamburg, Germany); Michel Morange (Institute for the History and Philosophy of Science and Technology (IHPST), Paris, France); Andrea Bertotti (Istituto di Ricovero e Cura a Carattere Scientifico (IRCCS), Candiolo, Italy); and Matthew Wood (University of Oxford, Oxford, UK).

Apart from the scientific symposium, a Career Day is foreseen, to allow participants to broaden their career perspectives. This activity will involve chats with professionals, high-quality workshops, and an Opportunity Fair, which will allow participants to come into direct contact with companies belonging to a variety of sectors. To support the participation of young researchers from all over Europe, our sponsors will provide about 40 travel grants to cover the registration fee and travel and accommodation expenses. Up-to-date information on the event can be found on our website (https://enablenetwork.eu/).

We look forward to the 2018 symposium and are confident that ENABLE will foster the establishment of a network that promotes efficient and synergistic scientific exchange among researchers throughout Europe.

